# Defining return-to-learn through an evidence-based systematic review

**DOI:** 10.3389/fneur.2026.1772377

**Published:** 2026-03-25

**Authors:** Zachary W. Bevilacqua, Joseph Fetta, Emily Vierno, Libby Cook, Kaitlyn Simone

**Affiliations:** 1 SUNY Brockport, Department of Kinesiology, Sport Studies & Physical Education, Brockport, NY, United States; 2 Department of Nursing, Quinnipiac University, Hamden, CT, United States

**Keywords:** concussion, definition, return to learn, return to school, return to class, RTL, RTLU

## Abstract

**Introduction:**

Academic recovery following concussion has been defined in various contexts, however, these definitions vary considerably leading to heterogenous data and limited application. Standardized definitions have also been published, though their formation are not transparently described. Therefore, this research aimed to report the various operational definitions of return-to-learn, return-to-school, return-to-class, and return-to-academics found within the literature and propose evidence-based definitions for return-to-learn (RTL).

**Methods:**

We searched PubMed and ScienceDirect for eligible studies that were (i) published in a peer-reviewed journal between January 2010, to January 2023, (ii) originally published in English, and (iii) had a full text available, (iv) measured RTL, return-to-school, return-to-the-classroom, or return-to-academics within a student sample, and (v) provided a clear definition or description of RTL completion criteria. Definitions of RTL, return-to-school, return-to-the-classroom, or return-to-academics along with outcome measure data (recovery times, group data, etc.) were extracted independently by three researchers and reached unanimous agreement.

**Results:**

The review yielded 456 articles, with 24 meeting inclusion criteria. Five recovery criteria were used across the studies: (i) guided protocols, (ii) clinician determinations/clearance, (iii) school attendance and days missed, (iv) resumption of full academic workload, and (v) full-time without accommodations. The proposed definition of RTL for college/universities is when the following criteria are met, (i) resolution of injury-associated symptoms, with and without academic engagement, (ii) resume pre-injury usage of accommodations, (iii) full academic participation—attend all registered courses, once minimum, attendance for the full time, (iv) cognitive functioning is stable and consistent with pre-injury baseline. The proposed definition of RTL for middle and high schools when the following criteria are met, (i) tolerates full academic demands without recurrence or worsening of injury-associated symptoms, (ii) has returned to their typical school schedule and workload, including extracurricular academic activities if applicable, (iii) no longer requires modified cognitive activity levels or informal classroom supports, (iv) demonstrates stable cognitive functioning consistent with their pre-injury baseline.

**Discussion:**

Academic recovery is defined by the literature in various ways, creating limited reproducibility of data. RTL definitions also display several gaps that do not account for logistical factors influencing return-to-learn across university and K-12 settings. Definitions lack a holistic approach, indicating that students may be prematurely satisfying recovery criteria. Novel definitions of recovery that are specific to the students individualized academic path, yet representative of the academic setting, are needed. The proposed definitions account for these variables, while remaining evidence-based.

## 1 Introduction

Return-to-Learn (RTL) has been investigated or sampled in some capacity for more than a decade; yet no definition has been widely endorsed to unify investigative efforts and support reproducibility of data. To complicate matters further, various terminology to describe academic recovery also exist. RTL, return-to-school (RTS), return-to-class, and return-to-academics have all been seen within the literature. Slight differences in language tempts confusion as terms suggestively correspond to specific stages of academic reintegration. For example, return-to-academics implies resuming any form of academic work [i.e., homework, step 2 (1)], whereas RTS denotes a return to the physical school setting [i.e., half day attendance, step 3 (1)]. Some data have been identified as RTS and return-to-academics but are defined by clinician determinations, not stages of a protocol (2, 3). The term RTL remains more ambiguous in its interpretation, with investigators assigning it to a range of operational definitions including the number of school days missed due to injury (4) and a resolution of injury-associated symptoms with class attendance (5, 6). Occasionally, the distinction between nomenclature is lost completely, for instance, “primary outcomes were… time until return to school, termed return to learn,” suggesting that terminology is interchangeable (4). The boundaries between operational terms has not been poignantly addressed, thus, the ability to draw sound conclusions from RTL data remains limited. For example, systematic review and meta-analysis found that RTL is achieved in a mean 8.3 days, though the recovery definitions of the included studies suffered from extensive discontinuity, reporting heterogeneous timeframes (1.53–24.75 days) (7).

Despite the current obstacle, interested parties will find some guidance from the recent RTL strategy put forth by the 6th Concussion in Sport Group consensus statement. The document promotes an RTL strategy, assigning language like RTS to the latter steps within the progression. This is a departure from the 5th consensus document which promoted an RTS strategy and used RTS to describe the various stages. It is conceivable that the 6th consensus document was purposefully revised to situate RTS as a step within an overarching RTL protocol, positioning RTL as the ‘endorsed’ term for complete academic recovery (1). This conclusion is furthered by the consensus documents’ addition of a dedicated RTL segment and a definition of RTL, which were absent from previous versions.

The 6th consensus authors define RTL as a “return to preinjury learning activities with no new academic support, including school accommodations or learning adjustments” (1). Moving forward, researchers have been encouraged to use this definition in hopes of improving reproducible and reliable RTL timelines. This definition appears prudent given its emphasis on re-establishing a pre-injury level of academic function with no reliance upon academic supports, yet the underpinning data and rationale remain unclear. Without this understanding, we will have limited ability to determine the quality of data produced from its criteria. For example, a recent systematic review of 32,766 articles uncovered that no published data have evaluated differences in kindergarten, elementary, or middle school student cohorts (8); yet consensus protocols have published academic re-integration protocols to be used by children, adolescents, and young adults (1, 9). Such a finding questions the true usability of an RTL protocol and its corresponding operational definition of recovery. Additionally, a criterion like a “return to preinjury learning activities” allows variability when determining whether “return” constitutes a preinjury form of engagement with learning activities, or re-engagement of any kind, which can undermine the standardized nature of the definition. To that end, this systematic review aimed to (i) report the various operational definitions of RTL, RTS, return-to-class, and return-to-academics found within the literature, (ii) discuss the characteristics of the compiled definitions, and (iii) consolidate this appraisal with evidence from RTL and concussion recovery as a whole to propose an alternative RTL definition to the 6th Consensus Statement that is age-specific, well-defined, transparent, and evidence-informed.

## 2 Materials and methods

### 2.1 Study design

The current review was formulated using a Population, Exposure, Comparison, Outcome, and Measure (PECOm) format (10). These variables are shown in Table 1 and established the research question, “How does concussion literature define academic recovery for university and K-12 students?.” The screening of data followed PRISMA guidelines (11).

**Table 1:** PECOm.

P	Population	K-12 and college students
E	Exposure	Concussion
C	Comparison	Published academic recovery/completion criteria
O	Outcome	Completed academic recovery
m	Measure	Full academic recovery

### 2.2 Search terms and databases

Search terms were selected using the confines of our PECOm, were agreed upon by all authors, and are presented in Supplementary materials 1. Search terms were entered into PubMed and ScienceDirect, and were restricted using the date range January 1, 2010, to 2023. The year 2010 was chosen as this represents when the first RTL focused paper was published (12). The PubMed search was conducted between January and March of 2023, whereas ScienceDirect was conducted in February of 2026. Articles were compiled by a single author (ZB).

### 2.3 Selection criteria

**Figure 1:**
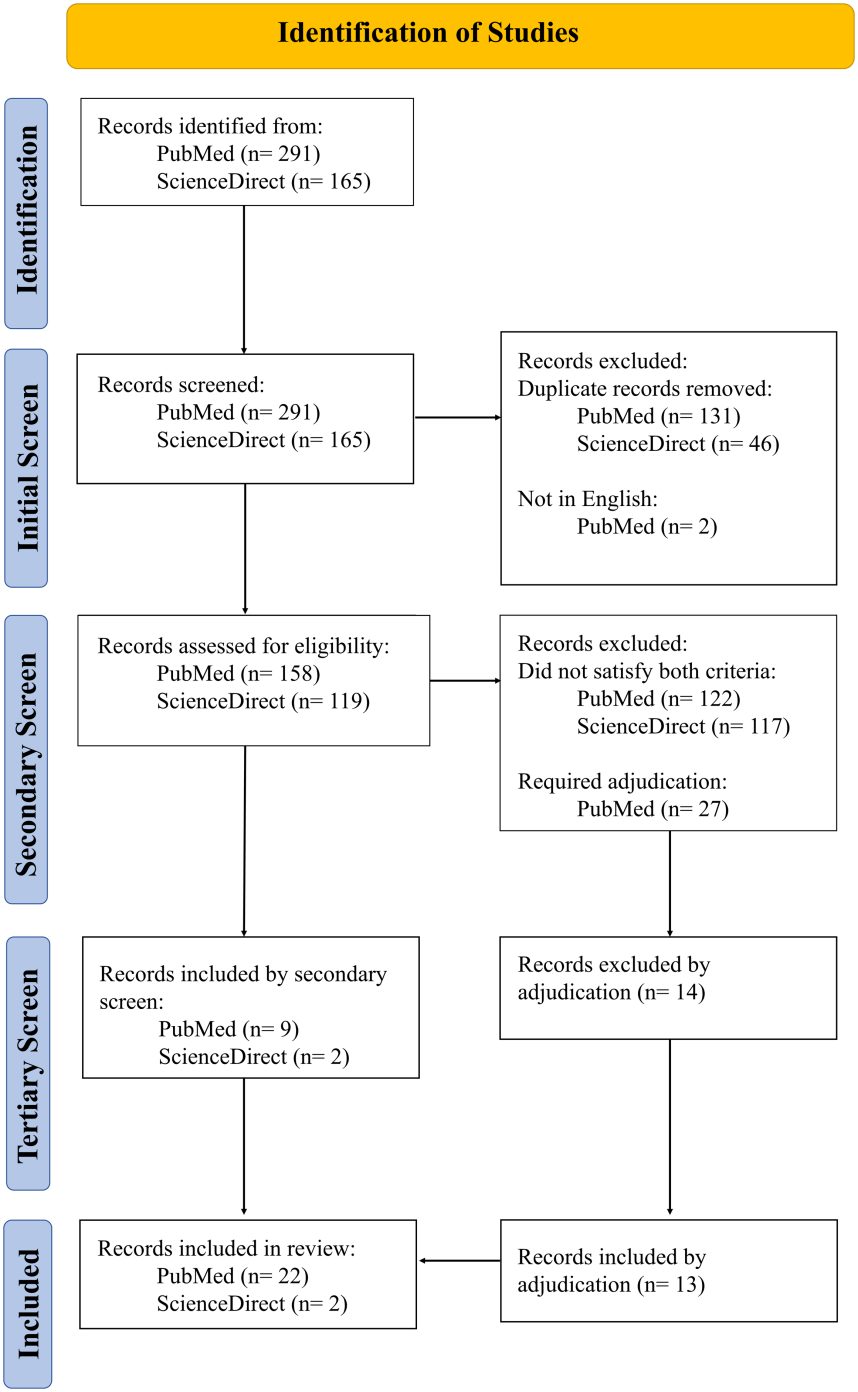
PRISMA flowchart.

All retrieved articles were subjected to a random initial screening by a pair of independent authors (EV, LC, KS) and a third adjudicator (ZB), if needed. Articles were retained if they were (i) published in a peer-reviewed journal, (ii) originally published in English, and (iii) had a full text available through open access or university library licensing. Duplicate articles were removed. Articles meeting all three criteria underwent a secondary screening (Figure 1).

Secondary screening was conducted by a randomly selected pair of independent authors (EV, LC, KS) and a third adjudicator (ZB), when needed. Secondary screening ensured that articles (i) measured RTL, RTS, return-to-the-classroom, or return-to-academics within a student sample, and (ii) provided a clear definition or description of academic recovery criteria. We elected to remove preconceived definitions of concussion as boundaries for inclusion to capture the full breadth of RTL papers and the recovery criteria therein. Thus, articles satisfying both criteria were included in data analysis (Figure 1).

### 2.4 Data extraction

Definitions of RTL, RTS, return-to-the-classroom, or return-to-academics were extracted to support the primary aims of the review and were independently appraised by a random selection of three researchers (EV, LC, KS, ZB), reaching unanimous agreement (Table 2). Additional data were collected to aid in the analysis of extracted definitions, such as participants’ characteristics (age, athlete/non-athlete, grade, etc.), sample size, study design, frequency of symptom evaluation, symptom scale used, time until recovery, and whether symptoms were used to inform recovery (Supplementary material 2).

**Table 2:** Summary of findings.

Article	Ages (Years)	Recovery term used	RTL definition	Time until RTL
Ahluwalia et al. (13)	5–23	RTL	Returning to school with or without accommodations	Early therapy group: median 17.5 days [8–20.75] Late therapy group: median 12 days [IQR 3.5–26.5] (*p* = 0.09)
Bevilacqua et al. (6)	18–26	RTL	48 h of class attendance and baseline symptoms	Mean 18.3 ± 7.7 (min: 10, max: 36)
Bretzin et al. (26)	College-aged (not specific)	Return to Academics	The time to return to academics was determined from the date the concussive injury occurred to the date the athlete returned to full academic participation	Total sample: median 8 days [IQR 3–15] Males: median 7 days [IQR 3–14] Females: median 9 days [IQR 4–17] 50.6, 26.7, and 11% had not returned to academics 7, 14, and ≥ 35 days post injury, respectively
Chrisman et al. (14)	5–14	RTS	Returning to school full-time	Youth (90%) returned to school by a median 9 days [IQR 2–6] (mean = 6.7)
Chu et al. (25)	8–18	RTA	Days between patient-reported date of injury to the date of medical clearance (i.e., asymptomatic unrestricted physical and cognitive activities for at least 3 consecutive days).	Males: median 26 days [IQR 18–39] Females: median 21 days [IQR 14–31]
Cook et al. (30)	14–19	RTS	Returned to school (full time without accommodations)	ADHD group: median 7 days [IQR 3–13] (range 0–45) Non-ADHD group: median 7 days [IQR = 3–13] (range 0–231)
Cook et al. (31)	14–19	RTS	Full-time without accommodations	Females: median 7 days Males: median 6 days (*p* = 0.33)
Corwin et al. (15)	5–18	RTS	Full-time return to school without academic accommodations (e.g., no homebound education, no breaks, regular testing without additional resources)	Vestibular deficits group: median 59 days Control group: median 6 days
DeMatteo et al. (16)	5–18	RTS	RTS Stage 5—Full Return to School	Total sample: median 35.3 days
Desai et al. (17)	7–18	RTS	Return to full school without accommodations	Females: median 4 days Males: median 3 days
Fisher et al. (18)	5–18	RTS	Completing Stage 5 of the RTS protocol, where participants resumed their normal (pre-injury) academic routine without symptoms	Total sample: median 56.5 days Early RTS group: median 35.6 days Delayed RTS group: median 100.6 days
Iverson et al. (27)	17–27	RTS	Returning fully to school without accommodations	Total sample: median 4 days [IQR 2–9] Low symptom burden group: median 3 days Medium symptom burden group: median 5 days High symptom burden group: median 6 days
Kenrick-Rochon et al. (28)	College-aged (not specific)	RTL	Returning to school without accommodations for class attendance, exams, or assignments	Median 17.5 days (2016–17) Median 6.5 days (2017–18)
Lawrence et al. (3)	15–20	RTS	Full return to school/work, as assessed or cleared by a physician	Total sample: median 23 days [IQR 15–36]
Martin et al. (19)	5–17	RTS	Return to academics without accommodations and achieving full baseline function as determined by clinic providers	Pre-existing anxiety group: median 83 days Control group: median 46 days
Martin et al. (20)	5–17	Return to Academics	Return to school or academics without accommodations, determined by clinic providers through graded return-to-learn protocols	Learning disorders group: median 58 days ADHD group: median 64 days Non-LD/ADHD group: median 49 days
Purcell et al. (21)	8–17	RTL	Return to school with or without accommodations	Patients aged 8–12 years: median of 4 days (range 0–30) 13–17 years: median 2.5 days (range 0–55.0) (*p* = 0.86) 60 and 71.8% of 8–12 and 13-17-year-olds RTL within 5 days of injury 72.9 and 73.3% of 8–12 and 13-17-year-olds RTL within 7 days of injury
Teel et al. (24)	5–17	RTS	Complete resolution of postconcussion symptoms and deficits, fully returned to school without accommodations, and performed moderate to vigorous cardiovascular exertion without symptom recurrence	Low stress group: median 40.5 days [IQR 28–49] Moderate/high stress group: median 55 days [IQR 34–81]
Terry et al. (22)	14–25	Return to Academics	Return to academics full-time without accommodations	Migraine history group: median 6 days [IQR 2–15] No migraine history group: median 5 days [IQR 2–9]
Waltzman et al. (32)	14–18	RTL	Return to full academic workload (self-reported)	Returned in less than 1 week = 42.7% Returned in 1–3 weeks = 44.1% Returned in >3 weeks = 13.2%
Wiebe et al. (29)	19–21	Return to Academics	Return to full academics	Total sample: median 6 days [IQR 2–13]
Wildgoose et al. (33)	Secondary school-aged (not specified)	RTL	Time until initial return to school & time until returning to school full-time	Time until initial return to school: Participant 1 = 2 weeks Participant 2 = 3 weeks Participant 3 = 2 weeks Participant 4 = 3 weeks Participant 5 = 2 weeks Time until returning to school full time Participant 1 = 4 months Participant 2 = 3 weeks Participant 3 = 1 month Participant 4 = 2 months Participant 5 = 1 month
Yengo-Kahn et al. (4)	12–23	RTL	RTL was measured as the number of missed school days (i.e., weekends not included) attributable to the injury and was not recorded if the SRC had occurred during the no-school summertime period (*n* = 3). An athlete was considered returned to school when either half or full days were resumed	White athletes: median 2 days [IQR 0–5] Black athletes: median 0 days [IQR 0–2] (*p* = 0.010)
Zuckerman et al. (23)	11.6–22.2	RTS	The number of school days missed	Total sample: median 2 days (range 0–90)

RTA, Return to Academics; RTL, Return to Learn; RTS, Return to School; SRC, Sport-Related Concussion.

## 3 Results

**Figure 2:**
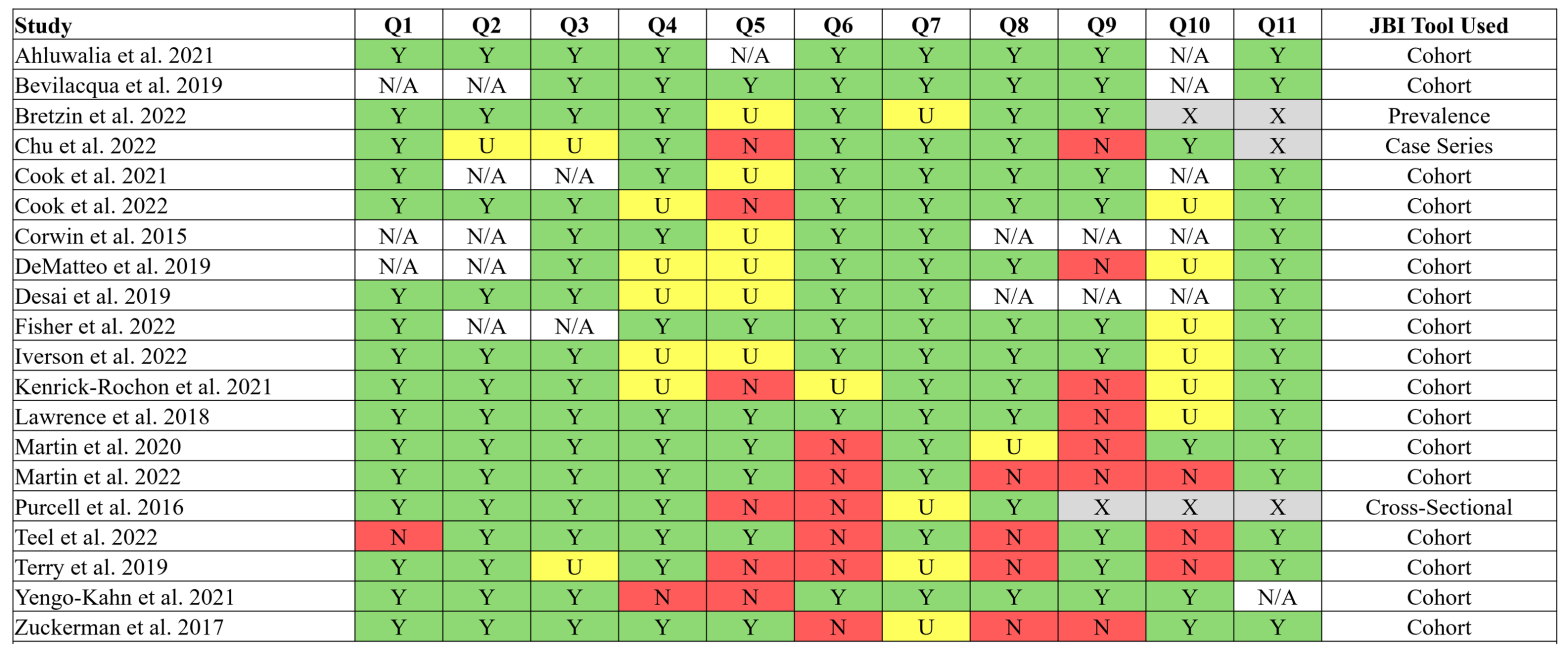
Joanna Briggs Institute (JBI) risk of bias assessment. Y, Yes; N, No; U, Unclear; N/A, Not applicable; X, Appraisal tool did not include 11 questions.

Twenty-four articles were included in the review (Figure 1). Fifteen articles reported on a mixed sample (children, adolescents, and/or university age) (3, 4, 13–25), five from university students (6, 26–29), and four from adolescents (30–33). No study presented data solely from a child-aged sample. Participant ages varied across studies despite belonging to similar age groups (i.e., children, adolescents, etc.), making age ranges difficult to define. Eleven articles collected data on sport-related concussion from athletes of varying ages (5–27 years) (3, 4, 13, 14, 22, 23, 25–29). Studies were largely retrospective reviews (*n* = 11) (3, 4, 13, 15, 17, 19–23, 25) and prospective cohort studies (*n* = 9) (6, 14, 16, 18, 27–31). Joanna Briggs Institute (34) risk of bias results can be found in Figure 2.

### 3.1 RTL definitions/criteria

**Figure 3:**
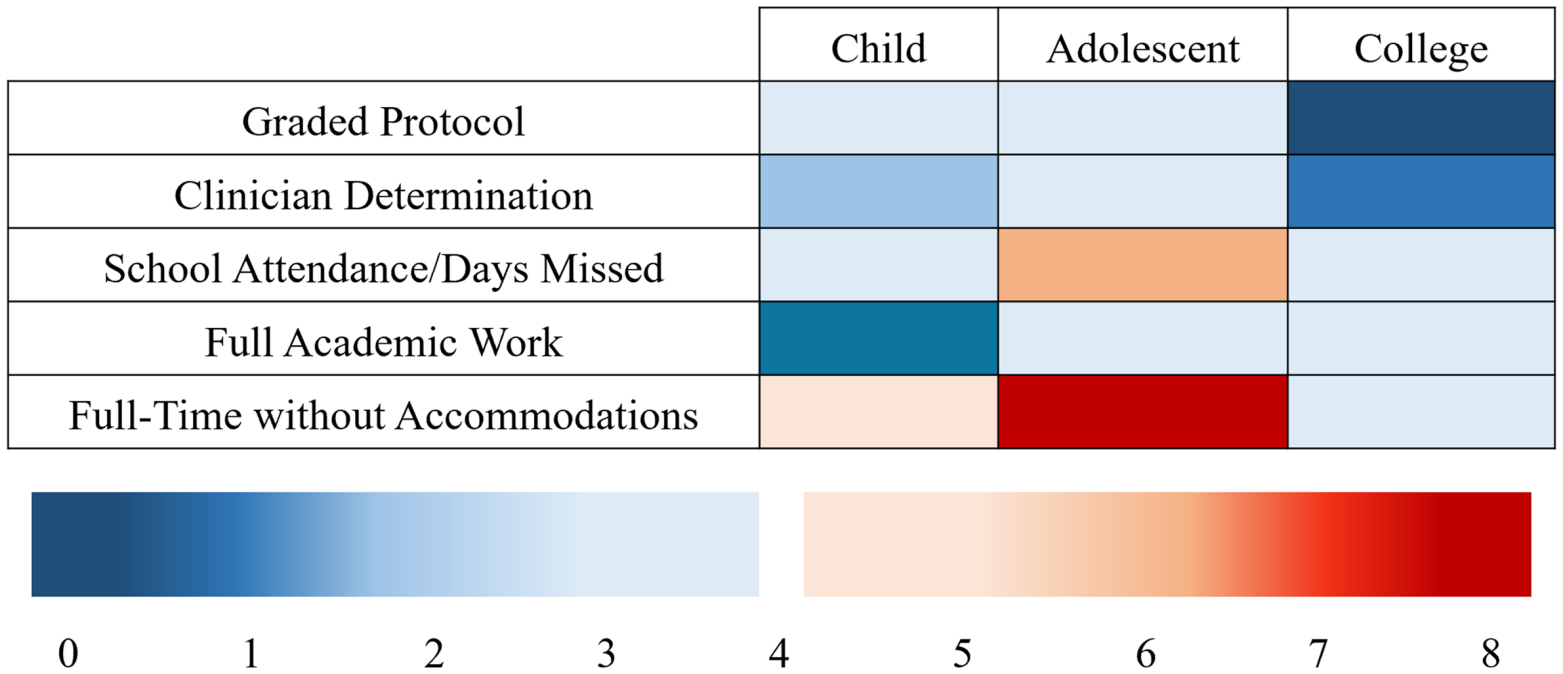
Heat map of recovery criteria vs. age group across included studies.

RTL definitions across the included studies displayed a diverse makeup. Three studies employed the completion of a graded protocol to assign full RTL, all of which were pediatric cohorts (16, 18, 20). RTL was also defined by clinician determinations (3, 19, 20), school attendance/number of days missed (4, 13, 14, 21, 23, 33), return to full academic workload (3, 25, 26, 29, 32), and 48 h of class attendance at pre-injury symptom levels (6); however, nearly half of the studies (*n* = 10) required a full return to school without accommodations (15, 17, 19, 20, 22, 24, 27, 28, 30, 31). Eight studies utilized symptoms to inform recovery (3, 6, 16, 18–20, 24, 25). Figure 3 visualizes definition criteria against age group.

### 3.2 Term used to define recovery

Five studies (20, 22, 25, 26, 29) termed recovery as return-to-academics, including college (26, 29), adolescent through college (22), and child through adolescent (20, 25) cohorts. Seven studies (4, 6, 13, 21, 28, 32, 33) used RTL, including college (6, 28), adolescent (32, 33), adolescent through college (4), child through college (13), and child through adolescent (21) samples. Twelve studies termed recovery as RTS, including adolescent (30, 31), adolescent through college (3, 27), child through college (23), and child through adolescent (14–19, 24) cohorts. No studies used return-to-class. RTS studies were comprised almost entirely of child (*n* = 8) and adolescent (*n* = 11) students, whereas RTL studies displayed more adolescent (*n* = 5) and college (*n* = 4) samples. Return-to-academics studies split between child, adolescent and college-aged cohorts.

### 3.3 Time until RTL

Collective median and mean RTL times ranged from 0 to 100.6 days and 6.7–18.3 days, respectively. Recovery in studies that did not use symptoms to inform RTL ranged between 0 and 231 days. Contrastingly, studies that did use symptoms ranged from 10 to 100.6 days. Median values were reported more often than mean [*n* = 22 vs. *n* = 2 (6, 14)]. Recovery data were reported from several samples: early therapy vs. late (13); low vs. medium vs. high symptom burden (27); female vs. male (17, 25, 26, 28, 31); Black vs. White (4); children vs. adolescents (21); early return to school vs. delayed (18); pre-existing anxiety vs. non (19); learning disorders vs. non (20); history of migraine vs. non (22); ADHD vs. non (30); vestibular deficits vs. non (15); low parental stress vs. high (24); and aggregate sample data (3, 6, 14, 16, 23, 29, 32, 33).

### 3.4 Symptom evaluation

Collection of symptom data varied greatly among the included studies. Many articles did not provide or were unclear with the frequency in which they collected symptom data (*n* = 13) (3, 13, 17, 21, 22, 24–26, 28–32), with others recording symptoms initially (*n* = 3) (15, 27, 33), every 48 h (*n* = 2) (16, 18), every visit (*n* = 2) (19, 20), weekly (*n* = 1) (14), four times per day (*n* = 1) (6), and at 3 months (*n* = 2) (4, 23). When symptoms were assessed, the Sport Concussion Assessment Tool (*n* = 6) (3, 14, 21, 29, 31, 33) and Post-Concussion Symptom Scale (*n* = 6) (13, 16–18, 22, 27) were utilized most often. Others chose the Acute Concussion Evaluation Inventory (*n* = 2) (19, 20), the Immediate Post-Concussion Assessment and Cognitive Test (*n* = 1) (15), Post Concussion Symptom Inventory (24), did not use a standardized symptom checklist (*n* = 1) (6), or were unclear in their approach (*n* = 6) (4, 23, 25, 26, 28, 30, 32). Several studies cataloged participant symptoms, yet few included this data as a component of their RTL recovery criteria (*n* = 8) (3, 6, 16, 18–20, 24, 25).

## 4 Discussion

This study aimed to report the various operational definitions of RTL, RTS, return-to-class, and return-to-academics found within the literature and subsequently provide age-specific definitions for RTL. The preponderance of studies contained a mix of age groups, reported on athletes, and followed retrospective methodologies. Furthermore, there was a lack of uniformity with the use of recovery terminology, representation of elementary school investigations, and most importantly, definitions of RTL or recovery. This could be a product of most studies (22, 24) using clinician determinations to diagnose participant concussions, potentially resulting in recovery criteria that were deemed relevant by the diagnosing clinician, practice, or hospital system. The various recovery criteria used across studies will be discussed, exploring their meaning and potential value within an RTL definition. Additionally, in keeping with consensus rationale, RTL will be used as the term representing full academic recovery.

### 4.1 Graded protocol

Multistage progressions have guided concussion recovery for many years, incrementally exposing patients to the stressors of their pre-injury environments. These stressors can be categorized as environmental (e.g., class attendance, lights, noise) and cognitive (e.g., assignments, notetaking). For example, changes to a student’s cognitive load is routinely suggested following concussion beginning with home-based work before transitioning to participation in partial school days. This is logical given the real-time component of class attendance that is not mimicked at home where students can stop to rest without falling behind. Once tolerable, the student will return to the classroom setting, at which time environmental stressors are considered. Stage 3 from DeMatteo et al. (16), along with the consensus RTL strategy (1) align in this recommendation. Suggesting environmental accommodations can translate to several outcomes, which means careful thought should be given when selecting such changes. Preferential seating, for instance, has been advised for students (35), however, this may mean placing the student further from the classroom front rather than closer to, depending upon the presence of any ocular accommodative deficits, far- or near-sightedness, or deficit found with the clinical assessment of cranial nerve III. The classroom environment will also vary greatly between student samples, with K-12 settings absent of large lecture halls commonly found on university campuses. These spatial differences in classrooms could have cervicogenic implications for the student who must maintain cervical rotation, extension or flexion to view the front of the classroom. It is difficult to tell whether graded protocols are intended for university use given the absence of adult studies utilizing such protocols. Additionally, the language included throughout CISG RTL stages better reflects K-12 academics, such as ‘back to school’, ‘schoolwork’, and ‘school day’, where terms like ‘class’, ‘lecture’, and ‘course schedule’ are often found describing university academics. One collegiate protocol does promote a similar stepwise approach (36), though the supporting literature is not disclosed, and given the overlap with our search parameters (i.e., PubMed database, published 2010–2020), is likely derived from pediatric samples, encouraging caution with use. Data from university stakeholders has accumulated, spurring recent RTL protocols for university students that do not follow a graded approach (37, 38), supporting the conclusion that current stepwise RTL progressions, intended or not, hold a pediatric focus.

### 4.2 Clinician determinations

Concussion diagnostic and recovery practices encourage clinicians to exercise their judgment to best care for the patient. The Sport Concussion Assessment Tool can provide useful information in the acute evaluation of suspected concussion, though authors acknowledge that concussion remains a diagnosis made by the healthcare provider (39). It is further recommended that clinicians select relevant functional outcomes and return-to-activity measures to guide recovery determinations (1). In turn, recovery times will vary across patients of similar ages given the presence of pre-existing conditions (e.g., ADHD, anxiety, learning disability) and the individualized supports that tailor to those conditions. This could explain the RTL data from Martin et al. who utilized clinician determinations to establish recovery, and reported dissimilar timeframes in 5–17 year olds with various disabilities (median 83d vs. 46d; pre-existing anxiety vs. no anxiety) (19) (median 49d vs. 64d; learning disability or ADHD vs. no learning disability or ADHD) (20). Clinician assessments have value in that they are tailored to the patient’s individual needs, though the specificity of this approach sacrifices a degree of generalizability, making comparisons between studies and patients difficult. Perhaps a lack of data generalizability is acceptable for RTL, as academic demands and environments (e.g., workload, course of study, social environments, academic pressures, parental/guardian/teacher oversight, etc.) can differ considerably between patients.

### 4.3 School attendance/number of days missed

We previously noted the interchangeable use of terms to describe ‘resumption of academics’ post-concussion. If interpreted literally, RTS would indicate a return to the physical school building, fitting well with school attendance as a recovery criterion, though not all studies used RTS in this way. Four studies used RTS to discuss participant recovery, but would define it as achieving full baseline function as determined by the clinician (19), full schoolwork with physician clearance (3), and completion of a 5-stage protocol (16, 18). Three pediatric-aged studies required participants to RTS and be free of accommodations (17, 30, 31), which is curious since academic accommodations for this population are not awarded immediately, and require that the student show difficulties in school for a period of weeks; thus the data from these studies likely undershot recovery (median days 3–7) and perhaps missed academic accommodations that were awarded in the weeks following participants’ exit from the study. School attendance was also interpreted differently, with some requiring any attendance (23, 33), partial days (4), or full-time school (14, 15, 30, 31). Together, RTS has shown to possess many meanings, and encourages the narrative that RTL literature is found wanting of consistency.

### 4.4 Full academic workload

Complete resumption of academic work is a primary goal of RTL, however, equating full academic workload between students becomes difficult, especially in post-secondary education where degrees of study differ significantly. For instance, STEM (Science, Technology, Engineering, Mathematics) degrees often include course sequences that have laboratories components, necessitating additional hours of attendance, preparation, and assessment. Comparatively, performing arts degrees more heavily focus on physical movement and may include performance-based or physical activity assessments. Both academic workloads, in part, stress the brain in a similar fashion (i.e., pre-frontal reasoning, oculomotor efficiency), though the unique requirements between students (cognitive vs. physical) must be considered when publishing aggregated university-level RTL data. Furthermore, academic demands increase as students progress from elementary school through to the terminal years of secondary education and beyond. Thus, studies that report data across multiple levels of academia, much like those included in this review (4, 13–20, 22, 23), will experience the same overgeneralization, given that it is unreasonable to assume that academic workloads and pressures are commensurate for a 7-year-old as they are for a high school senior.

Four studies required a return to academic baseline function, and present aggregate data across child and adolescent students (16, 18–20). Several other studies do not specify the academic workload needed for recovery, but similarly sum data between age groups (4, 13–15, 17, 22, 23, 25), with one exception (21). Authors have highlighted the aberrant gaps of RTL knowledge among K-8 students (8), along with the differences between academic settings (37, 40) (i.e., secondary school vs. university); thus, moving forward, RTL research should depart from aggregating data across multiple age groups and subscribe to separation of data by academic setting (elementary, middle school, high school, college).

### 4.5 Full-time school without accommodations

For K-12 students, full-time school implies attendance for the entire school day, though defining this for older students is not straightforward. For university students, ‘full-time’ could mean attending the day’s courses in full, or attending every course the student is registered for from start-to-finish. Considering the dispersed nature of university scheduling, attending each course could consume an entire 5-day week. Only two studies specified the amount of time needed to assign recovery for students, which were set to 48 h (6) and 3 days (25). The authors rationalized their criteria by stating how a 48-h period would limit false positives should symptoms return following a single day of class attendance and/or exposure to a limited variety of coursework (6). Otherwise, studies seem to pair full-time school with full-day attendance.

Numerous studies required students to be without accommodations prior to reaching recovery criteria (15, 17, 19, 20, 22, 24, 27, 28, 30, 31). The term ‘accommodations’ must be understood as a legally binding directive of agreed upon supports (e.g., extended time/delayed due dates, quiet testing area, notetaker, screen reader, etc.) to be carried out by faculty. K-12 schools will often refer to this as a ‘504 plan’, while higher education does not. Accommodations are fundamentally different from ‘academic adjustments’, which are used at the teacher’s discretion and can be discontinued for any reason. Academic adjustments are encouraged in K-12 RTL as students would otherwise need to display a prolonged and significant detriment to their academic abilities over time (i.e., weeks) for school administration to justify implementing a 504-plan; thus, academic adjustments are often enough to support recovery and are more practical for immediate implementation. The temporal delay with accommodations in K-12 is key and likely contributed as a modifying variable to the pediatric data discussed in this review. For example, recovery for child and adolescent cohorts were between a median 3–7 days (15, 17, 30, 31), yet these students could not feasibly expect to receive a 504-plan within this timeframe, suggesting that the data either undervalued recovery time or used incorrect terminology to define academic support. None of the included studies requiring students to be free of accommodations specified or tracked whether accommodations were received informally or through a standardized process (i.e., 504 plan, Campus Disability Services Office); therefore, moving forward, researchers must improve the accuracy by which this criterion is assessed. Authors should further be mindful of the terminology used to describe this criterion, ensuring that it represents the studied population. For instance, teachers in K-12 have been encouraged to use *‘academic adjustments’* to support students post-concussion instead of invoking a formal 504-plan (41, 42). On the other hand, research indicates that university students are better supported by formal accommodations issued through the Campus’ Accessibility/Disability Services Office (37, 40, 43); thus, *‘accommodations’* would be a more appropriate criterion for these samples. Uniquely, students who utilize accommodations pre-injury should not be expected to shed their existing supports to meet recovery criteria, infusing an additional layer of consideration when incorporating accommodations as part of an RTL definition.

### 4.6 Proposed definitions

Findings from this systematic review provided a strong footing in which to begin contemplation of a RTL definition that is well-defined, transparent, and evidence-informed; however, because RTL is multifaceted, parallel data was needed to construct and rationalize a definition that is age and setting-appropriate and can translate into practice. The proposed definitions are intended to reflect the scope of RTL including the various facets considered when determining academic recovery, such as: clinical symptom resolution and relapse management, functional academic stability, and pragmatic academic readiness.

#### 4.6.1 Return-to-learn for university students

Altogether, RTLU is achieved when the following are met:

1. *resolution of injury-associated symptoms, with and without academic engagement (home assignments, in-class work, course-associated demands)*
2. *resume pre-injury usage of accommodations*
3. *full academic participation—attend all registered courses, once minimum, attendance for the full time*
4. *cognitive functioning is stable and consistent with pre-injury baseline*

##### 4.6.1.1 Resolution of injury-associated symptoms, with and without academic engagement

Acute symptom burden remains the strongest predictor of recovery (44), yet a mere six studies included symptom resolution as part of recovery. Symptoms can result from an autonomic dysregulation of cerebral blood flow (45, 46), secondary axotomies like ion fluctuations (Na+, Ca++), calcium and calpain driven cell apoptosis, neurometabolic deregulation, glutamate excitotoxicity (46, 47), or uncoupled somatosensory function (vestibular-ocular, cervical-ocular). Additionally, symptom resolution timelines are aligning steadily more with neural recovery (i.e., 30 days) (1, 48). Changes to symptom profiles during functional activities (e.g., exercise, reading) are used as markers of this recovery, with a symptomatic response to a functional assessment suggesting that resolution of primary and secondary axotomies are still in progress. Sub-symptom threshold physical and somatosensory activities are known to hasten symptom resolution versus no intervention or placebo (49–53), indicating that no more than a mild and brief exacerbation of symptoms (1) with symptom-limited academic activity be a component of an RTLU path; however, given the predictive consistency between initial symptom burden and recovery time (44), resolution of injury-associated symptoms during unrestricted academic activity should contribute to an RTL ‘finish line’. Because initial symptom burden remains a significant prognosticator of recovery (1, 7), resolution of injury-associated symptoms both during and after unrestricted academic activity should minimally be incorporated into an RTLU definition. This criterion mimics return-to-play and is similar in its intent to protect students from premature clearance and removal of support. The CISG consensus definition and associated stepwise protocol for RTL do not include full resolution of injury-associated symptoms (1). This raises concerns, with 42% of sample college students prematurely returning to learn, experiencing more symptoms and becoming cognitively overwhelmed (54). Still, a university student reaching ‘recovered’ status without complete resolution of injury symptoms is worrisome and contradictory to established concussion treatment; thus, we maintain the inclusion of injury-associated symptom resolution as a marker of recovery.

##### 4.6.1.2 Resume pre-injury usage of accommodations

Accommodations were included in college-sample RTL definitions 41% of the time (27, 28), however, it remains unclear whether authors defined accommodations as an accessibility sponsored plan, or as something more informal. Collegiate athletes received “accommodations” 32% (26) and 48% (55) of the time, which could be a product of the structured sports medicine team surrounding these students. Anecdotes from the CISG document note that many students will return to academics with minimal difficulty or need for an RTL strategy and academic support (1). Suffice it to say, we can cautiously infer that few students who suffer a concussion will receive academic support. Consequently, most college students will resume pre-injury usage of accommodations immediately, which may have taken place in the included studies. This will likely remain the case until accommodation referrals weave their way into routine clinical practice, and has been highlighted as a pivotal component within novel evidence-based RTLU guidance (37). The considerable benefits of accommodations throughout RTLU have been promoted by university students (56–58), with a history of concussion increasing the odds of receiving education to use accommodations (OR = 22.5, CI: 2.32, 218.35) (55), together asserting accommodation monitoring into our definition. There again, emphasis with this criterion is placed on resuming pre-injury usage of accessibility sponsored accommodations.

##### 4.6.1.3 Full academic participation, attend all courses, attendance for the full time

The criterion of full academic participation aligns with requirements from nine included articles, two from college cohorts, though no quantity or type of academic engagement or attendance has been agreed upon to determine what constitutes full academic participation. Moreover, the framework of university academics makes arriving at an answer increasingly difficult. For example, a large university may offer upwards of 170 fields of study (59), consisting of coursework that ranges between 50 min to 3 h in length, offered from morning to evening. Such high variability suggests that the academic experiences for any pair of students will differ, hence the thought to use a common denominator for an RTLU definition. An objective footing for this decision is realized when factoring in neuroimaging data. For example, investigation of a 20-min psychomotor vigilance task reports increased cerebral blood flow in functional areas of attention (i.e., inferior frontal cortex, anterior cingulate cortex, bilateral basal ganglia, etc.) in acutely concussed adults (< 2 weeks post-injury) versus healthy controls (60). Increased cerebral blood flow during cognitive tasks is hypothesized to represent an augmented cerebral effort or compensatory recruitment, thought to manifest fatigue (60); thus, the demands of a 20-min task are theoretically worsened incrementally if extrapolated to a 50- or 180-min course. Assessing a student’s response to a variety of temporal demands would help clinicians null the uncertainty of concussion-related fatigue recurrence, underpinning the decision to require attendance for the full duration of all courses. The CISG definition requires a ‘return to preinjury learning activities’ (1), though similar temporal criteria are absent, inviting variable interpretation. Furthermore, given the scheduling of college courses throughout the week, attendance should not follow the progression from half-day to full-day found in the reviewed studies (16, 18) and CISG RTL strategy (1). Rather, students should use their discretion while achieving RTLU attendance requirements, expending cognitive energy on the most important courses within a given day.

Our criterion uniquely requires attendance for all courses, at least once. Magnetic resonance imaging data helps frame why the various academic demands from courses should be considered. Areas of working memory and attention (BA9, BA46, BA10) (61) are generally stressed by college courses, much like n-back tasks. Randomized controlled fMRI data reports increased activation of bilateral prefrontal cortices (BA46, BA10) during an n-back task immediately, 2 weeks and 2 months post-concussion in college athletes versus healthy controls (62). Diminished resting state connectivity has also been seen (BA9, BA46) in this population acutely following injury, versus uninjured controls (63). These data suggest that college-aged students can experience prefrontal cortical deficits, both at rest and with coursework, over the duration of their academic recovery (i.e., ≤ 2 months). More can be estimated when factoring in specific academic disciplines. Arithmetic operations, while thought to share connections with language, will diverge bilaterally to parietal regions in college-aged individuals when received in an auditory fashion, much like a college lecture (64). Scientific creativity is significantly associated to BA10 and 18, where artistic creativity associates with BA6 and 32 (65). College-aged individuals who play a musical instrument will excite connections within the sensorimotor network (BA4, BA3,1,2, BA6) and between right precentral and postcentral gyri (66). Taken together, these studies map the cortical regions utilized when attending general and content specific courses. By requiring attendance of all courses on a student’s schedule, clinicians will individualize functional recovery to their patients, thereby assessing whether neural connections within and between academically relevant cortical regions are able to manage tasks in an unrestricted fashion. Only one college-aged study quantified the required amount of class attendance (i.e., 2 days) (6), still, this may be insufficient to encompass a student’s array of courses. Lastly, heterogeneity across concussion injuries makes it difficult to predict which cortical regions were insulted or will interrupt academic engagement, sustaining the rational to employ this individualized albeit encompassing approach.

Finally, higher education courses introduce unique circumstances, such as online asynchronous degree programs, hybrid learning, clinical, residency, or internship education. It is recommended that these follow the same temporal requirements as in-person learning, with a 3-credit course requiring 75 min of engagement twice a week (Tues, Thurs), or 50 min three times per week (Mon, Wed, Fri). This is commensurate with a 3-credit in-person course. Clinical or experiential learning activities may impose separate demands (e.g., lifting patients, rapid bending or changes in position (orthostatic), operating equipment or machinery, etc.) which rely upon physiological mechanisms like autonomic regulation of cerebral blood flow, balance and coordination; therefore, recovery of physiological mechanisms may need to be considered as covariates with RTLU decisions.

##### 4.6.1.4 Stable cognitive functioning consistent with pre-injury baseline

Stable baseline cognitive functioning is a fundamental ‘finish line’ for RTLU, yet the ability to assess cognitive function in an adult student presents unique challenges. College students are autonomous learners without the same oversight found in K-12. Consequently, evidence of the student’s academic function such as their ability to tolerate reading and computer use, or exam grades must be self-reported. Cognitive loads like these can be dosed similarly to graded protocols (1, 16, 18), with out-of-class assignments and readings that precede in-class work and examinations; still, as discussed previously with participation and attendance, the student should self-select the work to be completed. Student self-reports of difficulties with these tasks can be used as evidence of cognitive function, though, clinic-based measures should occur in conjunction with patient intake. Assessments like immediate and delayed recall, serial 7 s, and dual task gait are found in the Sport Concussion Office Assessment Tool 6 (SCOAT6) (67). Positively, the SCOAT6 is able to support serial evaluation of the student’s cognitive function, though validation is forthcoming. Other cognitive tasks involving academic-like oculomotor and cognitive functioning such as the King Devick test could also be of value, demonstrating some prognostic qualities for RTLU (5) along with college-athlete normative values to support comparisons (68). Finally, it should be understood that merely engaging in a full academic workload, as required by four studies (3, 26, 29, 32) and the CISG RTL strategy (1), does not equate to restored cognitive function. Thus, the combination of clinic-based cognitive assessments, available academic evidence, and clinician-student dialog are suggested to support this recovery criterion.

#### 4.6.2 RTL for K-12 students

The proposed RTL definition for K-12 instruction utilizes components of the aforementioned RTL benchmark in alignment with the 6th consensus statement on concussion. The RTL process for K-12 education is defined as the gradual, individualized and injury-associated symptom-guided reintegration to academic activities following a concussive injury. This process will require a collaborative effort from healthcare professionals, teachers, counselors, administrators and athletic trainers to ensure a stepwise increase in cognitive demands with constant monitoring of symptom exacerbations. Successful RTL, or RTL completion would be measured by the return of the student to full attendance throughout the school day without the use of individualized academic supports and resolution of injury-associated symptoms. The preponderance of included studies (19/24) presented data from child and adolescent cohorts, mainly in aggregate (15/19). The proposition of criteria is made through combination of these data paired with current evidence in adolescent research and extrapolated to K-6 with neurodevelopmental literature and expert reasoning. We propose four criteria for successful RTL. It is achieved when the student:

i) *tolerates full academic demands without recurrence or worsening of injury-associated symptoms*
ii) *has returned to typical school schedule and workload, including extracurricular academic activities if applicable*
iii) *no longer requires modified cognitive activity levels or informal classroom supports*
iv) *demonstrates stable cognitive functioning consistent with their pre-injury baseline*

##### 4.6.2.1 Tolerates full academic demands without recurrence or worsening symptoms

The K-12 RTL process aligns with seven of the included child and adolescent studies with its necessary inclusion of injury-associated symptom resolution. The resolution of injury-related symptoms during reintegration and after unrestricted academic workload resumes should be a fundamental requirement in the K-12 RTL definition. Students should be allowed to resume academic activities while not experiencing more than a mild and brief exacerbation of symptoms; however, in alignment with the current return-to-play progression, injury-associated symptoms must resolve to satisfy this recovery criterion. A premature completion of RTL progression while still experiencing concussion symptoms presents significant educational and developmental concerns. Students may struggle with information processing, attention, memory consolidation, and executive functioning all essential for academic success (69). Additionally, 28–49% of primary and secondary school students will prematurely RTL (54, 70), further cautioning the acceptance of a symptomatic RTL completion. In this way, our criterion differs from recent CISG RTL recommendations (1).

##### 4.6.2.2 Returned to typical school schedule and workload

The K-12 RTL aligns with five included studies (3, 25, 26, 29, 32), with specific attention to the setting in which students are expected to perform. Complete attendance across all classes on a student’s K-12 schedule serves as the definitive measure of cognitive readiness, as clinicians must individualize student recovery to account for simultaneous cognitive demands inherent in elementary, middle, or high school curricula. Specific attention must be paid to each individuals’ variable schedule and the cognitive load of 6–8 unique subjects each day. Gradual re-introduction to a student’s typical schedule can effectively support the integrated academic processing required for all educational activities. Additionally, the need for complete return to a typical schedule and workload prioritizes recovery of neural connections within and between relevant cortical regions over an individualized period. Lastly, schools will vary in their structuring of the academic day, sequencing classes on a recurring alphabetical or numerical schedule (i.e., ABCD schedule, Day 1, 2, 3). Therefore, students may need to attend multiple full days of school to experience the range of their schedule, made explicit by only a single included study (25). As outlined by three studies (16, 18, 20), resumption of the school schedule and workload can be successfully completed through a gradual stepwise approach.

##### 4.6.2.3 No longer requires modified cognitive activity levels or informal classroom supports

Eight studies required pediatric samples to be without accommodations in order to achieve recovery (15, 17, 19, 20, 22, 24, 30, 31); yet, none of these outlined how accommodations were received or monitored, constraining the utilization of these studies as an evidentiary basis for the proposed criterion. However, clinical reasoning and available consensus literature serve as strong replacements. Routine K-12 RTL progressions does not require formal academic accommodations such as 504 Plans or IEPs, but rather relies on flexible classroom strategies and *‘academic adjustments’* that can be implemented informally by educators during the recovery period (41, 71). Formalizing a 504-plan through the ADA standards leads to an unrealistic expectation of instituting supports prior to anticipated symptom resolution and full academic resumption timelines (1, 48). This, coupled with the legal requirement of only a singular annual review of 504 plan makes formal accommodations an unnecessary and perhaps a detracting component to a K-12 RTL definition. Instead, the proposed definition uses informal academic supports in place of legal accommodations through section 504 of the ADA, which should similarly revert to pre-injury use when determining RTL status. For those students with a pre-existing 504 plan, usage of accommodations should resume pre-injury levels as well. Formal evaluation of pre-existing accommodations through the ADA (504 or IEP) do not require adjustment or review during the recovery phase, however, regular evaluation of 504/IEP plans would continue to take place within the standard protocol of each school district. Implementation of additional academic support through K-12 RTL would be in addition to existing plans and not a permanent fixture due to the typical recovery timeline. This aligns with the consensus definition of successful RTL in that recovery should exhibit a removal of any new, or injury-necessitated academic supports.

##### 4.6.2.4 Demonstrates stable cognitive functioning consistent with baseline

The K-12 RTL process also necessitates the ability to demonstrate stable cognitive functioning for successful RTL. Diffusion imaging studies in adolescents provide evidence supporting microstructure changes in white matter tracts associated with cognitive and executive functioning necessary for academic success persisting greater than 3 months, despite a lack of clinical symptom experience (72–76). Specifically, there were diffusion abnormalities, functional hyperconnectivity and decreased choline along the superior longitudinal fasciculus and cortical connection white matter tracts after 3 months post injury (74). Studies using cognitive tasks similar to classroom activities including working memory, task switching, and attention found lower fractional anisotropy and higher mean diffusivity in frontal and temporal tracts (77). These diffusion changes were associated with poorer performance on these tasks, suggesting that diffusion abnormalities have relevance to school-related cognitive demands (77). Additionally, K-12 education requires students to readily access previously learned knowledge and continue to build upon it daily. Cognitive fatigue from frequent retrieval can be a detrimental shortfall for students who are still mending these essential white matter tracts. Finally, persistent diffusion changes in the frontal and parietal white matter tracts of children are associated with ongoing neurocognitive and neuropsychiatric symptoms, suggesting that these areas are particularly vulnerable and relevant for RTL decisions (69). Research also demonstrates a moderate and significant relationship between executive functions and academic performance in primary education (69). Because children and adolescents are in critical developmental windows, cognitive instability can create disruption throughout the school day. For example, when cognitive functioning is unstable, students experience unpredictable academic performance that can lead to learned helplessness and academic anxiety lasting for years beyond injury-associated symptom recovery (69); therefore, we find stable cognitive functioning to be a necessary component to an RTL definition. This will also support the reintegration of students to the academic setting and numerous social interactions necessary for development in the formative years of K-12 education.

Baseline cognitive functioning will expectedly be assessed clinically using mixed methods depending upon the student’s classes and extracurricular activities (e.g., band, theater), and would improve with input from the student, teachers, school counselor, school nurse, diagnosing provider, and parent(s)/guardian(s). These recommended team members (8) can offer perspectives of varying objectivity to best illustrate the student’s cognitive function. Pediatric studies in this review reflect this process of determining recovery through clinician assessment (3, 19, 20). Furthermore, the Child SCOAT6 (78) offers cognitive assessments to support clinical evaluation. Reference data for the King Devick test also exists in both child (79, 80) and adolescent (80) athlete populations, displaying high internal consistency, and across age ranges.

### 4.7 Limitations

The overall lack of homogeneity among studies can be interpreted as a limitation, emphasizing the need for consolidation and standardization of terminology to strengthen RTL protocols in the future. Variability in study design, population and outcome measures emphasize the need for consolidation of terminology as well as definition of RTL to eliminate future inconsistencies in research. Additionally, the reporting of median values for RTL is limiting as this does not account for the number of students on either end of the spectrum; therefore, mean and range data should be included to better visualize RTL and RTLU data. Lack of outliers in both arenas of RTL implementation does not capture the need for individualized RTL to ensure those impacted are afforded the same opportunity. None of the included studies reported on elementary population in isolation, thus, the applicability of the proposed K-12 RTL definition in these cohorts remains questionable. While the suggested definition would be applicable to elementary aged students, the definition of K-12 RTL and associated terminology will require further study in explicitly K-6 aged cohorts for efficacy of RTL with this suggested definition and criteria. This, coupled with the relative dearth of data on younger students reduces the overall generalizability to the elementary-aged populations. Additionally, elementary-aged children may face greater challenges with respect to developmental stages and cognitive deficits. Finally, our review included databases that encompassed medical, education, social, and biomedical sciences, however, we were unable to include other pertinent databases like Scopus due to subscription barriers, which could have limited the number of studies meeting inclusion criteria.

## 5 Conclusion

This review proposes definitions for K-12 RTL and RTLU through a systematic and transparent appraisal of the literature. Academic recovery was defined by the literature in various ways, using criteria like school attendance, number of days missed, accommodation usage, clinician determinations, and completion of a graded protocol. The definitions of K-12 RTL and RTLU are encouraged for use given their specificity to differing academic environments and expectations. By explicitly considering the unique challenges present in both K-12 and university settings, the proposed definitions acknowledge the complexity of educational trajectory, developmental stages and highly variable environments. This flexibility allows each definition to serve as a practical guide for clinicians, educators and administrators to support individual students’ reintegration to full academic workload while minimizing the risks of premature return, injury-associated symptom exacerbation and potential educational setbacks. Establishing this unified framework lays a foundation for consistent protocol development, informed policy, and individualized accommodations throughout the educational spectrum.

## Data Availability

The original contributions presented in the study are included in the article/Supplementary material, further inquiries can be directed to the corresponding author.
