# Supplementary material for: Defining return-to-learn through an evidence-based systematic review
Source: Front Neurol. 2026 Mar 25;17:1772377. doi: 10.3389/fneur.2026.1772377 (PMC13056662; doi:10.3389/fneur.2026.1772377)
Supplement: Supplementary file 1 [file Table_1.docx]

| **Supplemental Material 1.** |  |
| --- | --- |
| **Search Terms [PubMed]** | Results |
| ((concussion[Title/Abstract]) AND (return to learn*[Title/Abstract])) AND (colleg*[Title/Abstract]) | 17 |
| ((concussion[Title/Abstract]) AND (return to learn*[Title/Abstract])) AND (university[Title/Abstract]) | 6 |
| ((concussion[Title/Abstract]) AND (return to learn*[Title/Abstract])) AND (k-12[Title/Abstract]) | 0 |
| ((concussion[Title/Abstract]) AND (return to school[Title/Abstract])) AND (colleg*[Title/Abstract]) | 8 |
| ((concussion[Title/Abstract]) AND (return to school[Title/Abstract])) AND (university[Title/Abstract]) | 2 |
| ((concussion[Title/Abstract]) AND (return to school[Title/Abstract])) AND (k-12[Title/Abstract]) | 0 |
| ((concussion[Title/Abstract]) AND (return to class[Title/Abstract])) AND (colleg*[Title/Abstract]) | 0 |
| ((concussion[Title/Abstract]) AND (return to class[Title/Abstract])) AND (university[Title/Abstract]) | 0 |
| ((concussion[Title/Abstract]) AND (return to class[Title/Abstract])) AND (k-12[Title/Abstract]) | 0 |
| ((concussion[Title/Abstract]) AND (return to classroom[Title/Abstract])) AND (colleg*[Title/Abstract]) | 1 |
| ((concussion[Title/Abstract]) AND (return to classroom[Title/Abstract])) AND (university[Title/Abstract]) | 1 |
| ((concussion[Title/Abstract]) AND (return to classroom[Title/Abstract])) AND (k-12[Title/Abstract]) | 0 |
| ((concussion[Title/Abstract]) AND (return to academ*[Title/Abstract])) AND (colleg*[Title/Abstract]) | 7 |
| ((concussion[Title/Abstract]) AND (return to academ*[Title/Abstract])) AND (university[Title/Abstract]) | 2 |
| ((concussion[Title/Abstract]) AND (return to academ*[Title/Abstract])) AND (k-12[Title/Abstract]) | 0 |
| ((concussion[Title/Abstract]) AND (return to school[Title/Abstract])) AND (adult[Title/Abstract]) | 1 |
| ((concussion[Title/Abstract]) AND (return to school[Title/Abstract])) AND (pediatric[Title/Abstract]) | 32 |
| ((concussion[Title/Abstract]) AND (return to learn*[Title/Abstract])) AND (adult[Title/Abstract]) | 2 |
| ((concussion[Title/Abstract]) AND (return to learn*[Title/Abstract])) AND (pediatric[Title/Abstract]) | 17 |
| ((concussion[Title/Abstract]) AND (return to learn*[Title/Abstract])) AND (student[Title/Abstract]) | 46 |
| ((concussion[Title/Abstract]) AND (return to learn*[Title/Abstract])) AND (adolesc*[Title/Abstract]) | 35 |
| ((concussion[Title]) AND ((systematic review[Title]) AND (return to class*[Title/Abstract])) | 0 |
| ((concussion[Title]) AND ((systematic review[Title]) AND (return to academ*[Title/Abstract])) | 1 |
| ((concussion[Title]) AND ((systematic review[Title]) AND (return to learn*[Title/Abstract])) | 1 |
| ((concussion[Title]) AND ((systematic review[Title]) AND (return to school[Title/Abstract])) | 5 |
| ((concussion[Title/Abstract]) AND (return to learn*[Title/Abstract])) AND (recover*[Title/Abstract]) | 46 |
| ((concussion[Title/Abstract]) AND (return to school[Title/Abstract])) AND (recover*[Title/Abstract]) | 53 |
| ((concussion[Title/Abstract]) AND (return to class[Title/Abstract])) AND (recover*[Title/Abstract]) | 0 |
| ((concussion[Title/Abstract]) AND (return to classroom[Title/Abstract])) AND (recover*[Title/Abstract]) | 2 |
| ((concussion[Title/Abstract]) AND (return to academ*[Title/Abstract])) AND (recover*[Title/Abstract]) | 6 |
| Total Results | 291 |
|  | |
| **Search Terms [ScienceDirect]** | Results |
| "concussion" AND "return to class" | 4 |
| "concussion" AND "return to classroom" | 1 |
| "concussion" AND "return to learning" | 11 |
| "concussion" AND "return to learn" | 44 |
| "concussion" AND "return to school" | 94 |
| "concussion" AND "return to academics" | 11 |
| "concussion" AND "return to academia" | 0 |
| Total Results | 165 |
