# Supplementary material for: Defining return-to-learn through an evidence-based systematic review
Source: Front Neurol. 2026 Mar 25;17:1772377. doi: 10.3389/fneur.2026.1772377 (PMC13056662; doi:10.3389/fneur.2026.1772377)
Supplement: Supplementary file 2 [file Table_2.docx]

| Supplemental Material 2. Additional Article Data | | | | | | |
| --- | --- | --- | --- | --- | --- | --- |
| Article | **Sample** | **Sample Size** | **Study Design** | **Frequency of Symptom Evaluation** | **Symptom Scale Used** | **Were Symptoms Used to Inform RTL** |
| Ahluwalia et al. 2021 | Sport-related concussion patients | n = 23 | Retrospective cohort study | Unknown | PCSS | No |
| Bevilacqua et al. 2019 | College students | n = 9 | Longitudinal observational study | Four times per day | 0–10 scale for five symptoms: headache, dizziness, difficulty concentrating, fatigue, and anxiety | Yes |
| Bretzin et al. 2022 | College varsity and club sport athletes | n = 1,974 (*498 lost to follow-up) | Descriptive epidemiology study | Unclear | Unclear | No |
| Chrisman et al. 2019 | Youth American football athletes | n = 863 were followed; n = 51 concussions occurred | Prospective cohort study | Weekly | SCAT3 | No |
| Chu et al. 2022 | Children and adolescents with SRC | n = 655 (362 F, 293 M) | Retrospective Case Series | Unclear | Unclear | Yes |
| Cook et al. 2021 | Adolescent student athletes | n = 623 | Prospective observational cohort study | Unknown | Unclear | No |
| Cook et al. 2022 | Adolescent student athletes | n = 375 | Prospective observational cohort study | Unknown | SCAT3 and SCAT5 | No |
| Corwin et al. 2015 | Pediatric patients | n = 247 | Retrospective cohort study | Initially | ImPACT | No |
| DeMatteo et al. 2019 | Children and adolescents | n = 139 | Prospective cohort study | Every 48 hours | PCSS | Yes |
| Desai et al. 2019 | Pediatric and adolescent patients with SRC | n = 192 | Retrospective cohort study | Specific intervals for symptom evaluation are not explicitly mentioned | PCSS | No |
| Fisher et al. 2022 | Adolescents and children | n = 94 | Prospective cohort study | Every 48 hours | PCSS | Yes |
| Iverson et al. 2022 | NCAA Division III collegiate athletes | n = 808 | Prospective naturalistic observational cohort study | Symptoms were evaluated within the first 72 hours post-injury | PCSS | No |
| Kenrick-Rochon et al. 2021 | University varsity athletes from eight teams across two academic years (2016–2017 and 2017–2018) | n = 30 | Observational cohort study | Reassessments typically occurred once every seven days, though frequency varied based on symptoms and availability | Concussion Recognition Tool 5 | No |
| Lawrence et al. 2018 | Individuals with acute sport-related concussions | n = 253 | Retrospective cohort study | Unknown | SCAT3 and SCAT5 | Yes |
| Martin et al. 2020 | Children and adolescents | n = 637 | Retrospective cohort study | Initial visit and all follow-up visits | 28-item dichotomous questionnaire adapted from the Acute Concussion Evaluation Inventory | Yes |
| Martin et al. 2022 | Children and adolescents | n = 680 | Retrospective cohort study | Initial visit and all follow-up visits | 28-item dichotomous questionnaire adapted from the Acute Concussion Evaluation Inventory | Yes |
| Purcell et al. 2016 | Children and adolescents | n = 198 | Retrospective cohort study | Unclear | SCAT2 | No |
| Teel et al. 2022 | Children and adolescents with concussion | n = 49 (27 F, 22 M) | Qualitative interviews | Not explicit, but performed at discharge | Post-Concussion Symptom Inventory | Yes |
| Terry et al. 2019 | High school and college athletes | n = 1,265 (485 high school, 780 college) | Retrospective cohort study | Unclear | PCSS | No |
| Waltzman et al. 2020 | Private high school students | n = 1,999 | Cross-sectional survey study | Unclear | Unclear | No |
| Wiebe et al. 2022 | College athletes | n = 1,715 | Prospective cohort study | Unclear | SCAT5 | No |
| Wildgoose et al. 2022 | Secondary school students | n =10 females | A grounded theory approach using in-depth qualitative interviews | Initial interview | SCAT3 | No |
| Yengo-Kahn et al. 2021 | Middle school, high school, & collegiate student-athletes | n = 247 (36 Black, 211 White) | Retrospective cohort study | 3 months post-injury | Unclear | No |
| Zuckerman et al. 2017 | Middle school, high school, & collegiate student-athletes | n = 282 | Retrospective cohort study | 3 months post-injury | Unclear | No |
| ImPACT = Immediate Post-Concussion Assessment and Cognitive Testing; PCSS = Post-Concussion Symptom Scale; SCAT = Sport Concussion Assessment Tool; SRC = Sport Related Concussion. | | | | | | |
